# Unique magnetostriction of Fe_68.8_Pd_31.2_ attributable to twinning

**DOI:** 10.1038/srep34259

**Published:** 2016-09-30

**Authors:** Jake Steiner, Abdellah Lisfi, Tomoyuki Kakeshita, Takashi Fukuda, Manfred Wuttig

**Affiliations:** 1 University of Maryland, Department of Materials Science and Engineering, College Park, MD 20902, USA; 2 Morgan State Univeristy, Department of Physics, Baltimore, MD 21251, USA; 3 Osaka University, Department of Materials Science and Engineering, Suita, Osaka 565-0871, Japan

## Abstract

Fe_68.8_Pd_31.2_ exhibits an anomalously large magnetostriction of ~400 ppm at room temperature as well as linear, isotropic, and hysteresis free magnetization behavior. This near perfectly reversible magnetic response is attributable to the presence of a large number of premartensitic magnetoelastic twin clusters present in the system made possible through the elastic softening that occurs near a martensitic transformation temperature of 252 K. It is proposed that the twin clusters in the material reduce both internal elastic and magnetic energy, causing the elastic and magnetic behavior of the material to be intimately linked. In such a framework, the anisotropy energy becomes extremely low causing the material to bear no crystalline dependence on magnetization, and application of a magnetic field causes simultaneous magnetic and twin domain movement which relaxes the system.

Magnetoelastic materials, those that exhibit a large change in shape in response to a magnetic field, are of significant interest in development of multi-ferroic technologies, such as ferromagnetic and ferroelectric composites, sensors, actuators, and cooling applications^1^,^2^,^3^,^4^,^5^,^6^,^7^,^8^,^9^,^10^. In these applications, a large deformation under a small applied field is desired along with low hysteresis. Well-known materials, like Terfenol-D, exhibit these properties, demonstrating near 2000 ppm strain when magnetically saturated at room temperature^1^. The discovery of ferromagnetic shape memory alloys (FSMAs) in 1984^11^ and subsequent research has revealed that these materials possess large deformations, close to 2000 ppm in the case of Ni_2_MnGa^12^, ppm when magnetically saturated. Their anisotropy energies are low, and very little hysteresis exists in the premartensitic range^3^,^13^,^14^,^15^. More recently, it has been shown that large, hysteresis-free magnetostriction in FeGa and related alloys is a phenomenon related to the microstructure of the alloy^16^,^17^. Here, we report on experimental results demonstrating a similar response in premartensitic Fe_68.8_Pd_31.2_. When decreasing the temperature, we demonstrate that the magnetostriction rises gradually in the austenitic state up to values of 400 ppm before undergoing a transition to a pseudo-orthorhombic state that adapts the cubic and martensitic states to each other. Thermal expansion results are used to corroborate the transformation temperatures. Using magnetic torque measurements, we show that the austenitic state is two-phase which explains the magnetoelastic nature of the demagnetized state.

## Results

### Thermal Expansion

**Figure 1:**
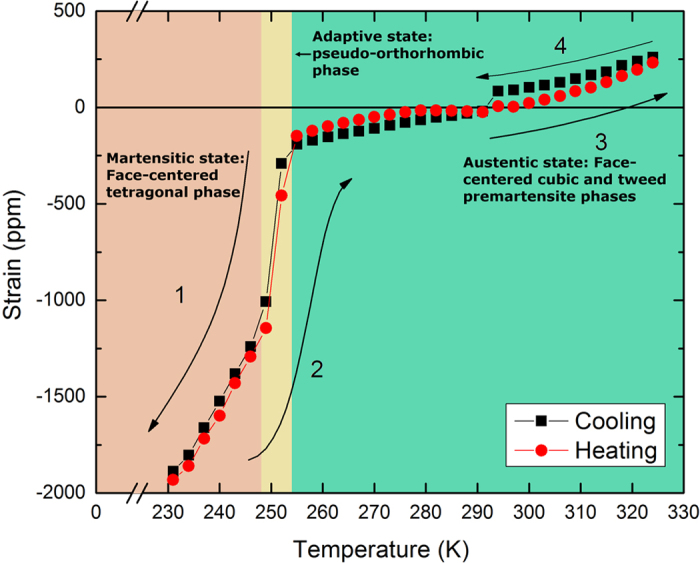
Thermal expansion data of with the strain gauge placed along a [100] direction for single crystal Fe_68.8_Pd_31.2_ with phases in austenitic and martensitic state labelled. The arrows and numbers dictate the flow of the thermal cycle, first decreasing down to 230 K from room temperature, followed by heating to 325 K then cooling to room temperature.

The thermal expansion of Fe_68.8_Pd_31.2_ is shown in Fig. 1. The abrupt change in strain between 252 K and 249 K signals a martensitic phase transformation in the alloy. This transformation in Fe_68.8_Pd_31.2_ has been documented as one from face-centered cubic (FCC) to adaptive pseudo-orthorhombic crystalline state to a martensitic face-centered tetragonal (FCT) state^18^,^19^,^20^,^21^. The FCC state possesses a unique microstructure of modulated nanoscopic twin precursors called premartensitic tweed, the nature of which is still not entirely concrete, though the consensus points to this tweed as a form of nuclei which coalesce before the martensitic transformation temperature, similar to a second-order phase transformation mechanism^20^. We propose this tweed forms the basis of a second phase in the austenitic state despite not appearing through diffraction experiments in past studies, and that it forms the basis for many of the unique properties found in Fe_68.8_Pd_31.2_. Among these, Fe_68.8_Pd_31.2_ exhibits an invar effect over a small temperature region, 280–295 K. This invar effect has been reported before in an FePd alloy of similar composition^22^, and the effect can be attributed to coherent modulated structures accomodating thermal strain such as has been observed in Fe-Ni^23^. The small jump seen in the cooling data at 293 K is a small error due to instrumentation.

### Magnetization

**Figure 2:**
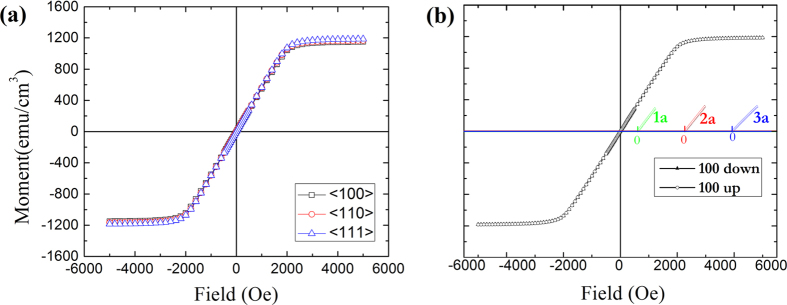
Magnetization curves taken at 293 K for (**a**) several crystal directions and (**b**) over several hysteresis loops, 1a, 2a, and 3a, of increasing (up) and decreasing (down) field, offset for clarity, for single crystal Fe_68.8_Pd_31.2_.

In Fig. 2 we present the results of magnetization measurements carried out on Fe_68.8_Pd_31.2_ at 293 K. The behavior is linear approaching saturation, possesses no hysteresis, and is independent of crystalline direction. The coercive force is very small and measures 0.05 Oe^24^. In Fig. 2b, we demonstrate that over the course of several magnetization loops the behavior remains unchanged, meaning the material exhibits near perfect reversibility. Though not shown, the response is independent of temperature from 360 K down towards the transformation temperature at 252 K. Typically, a magnetic response such as this reflects a mechanism by which all ferromagnetic domains in the sample are pinned, meaning the spins can only rotate towards the applied field, a fact which could only be true if the material lacked crystallinity, e.g. metglass. This is not the case for Fe_68.8_Pd_31.2_. Instead, the evidence points towards an isotropic demagnetization field, and we propose this is a consequence of ultra-low anisotropy caused by the manifestation of premartensitic nano-twinned clusters as the material becomes elastically soft approaching a face-centered cubic (FCC) to face-centered tetragonal (FCT) transformation^18^.

### Magnetic torque

**Figure 3:**
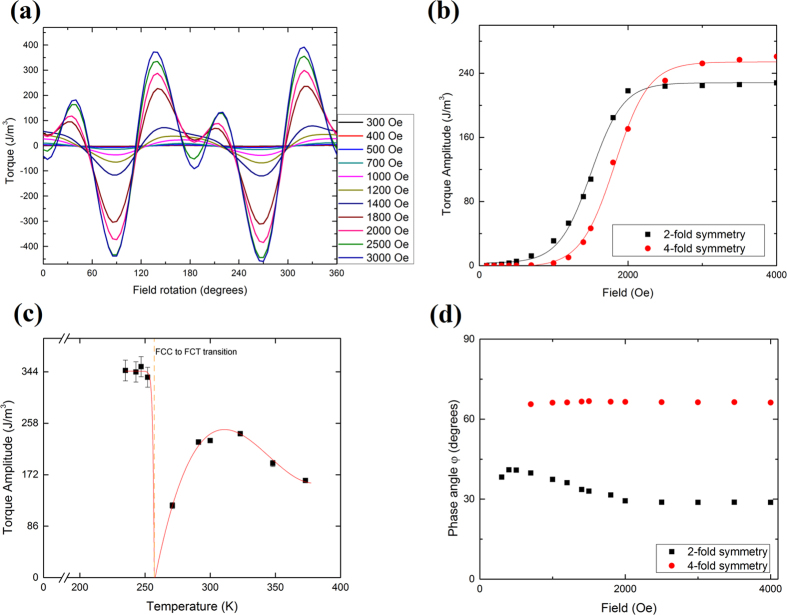
(**a**) Magnetic torque data of several field strengths taken at 293 K for single crystal Fe_68.8_Pd_31.2_, (**b**) change of torque amplitudes *A* and *B* fitted to 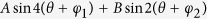 for curves of (**a**) to separate the anisotropy behavior of the ◼ 2-fold symmetry and ⚫ 4-fold symmetry, (**c**) temperature dependence of the 2-fold symmetry with applied field of 3000 Oe, and (**d**) change in phase angles *φ*_1_ and *φ*_2_ as a function of field for curves in (**a**).

The results of magnetic torque measurements, shown in Fig. 3a, indirectly support the possibility that magnetization is achieved through a twinning mechanism. From the data, the magnetocrystalline anisotropy energy density equals less than 10^2^ J/m^3^ in the linear region and is about 425 J/m^3^ at saturation, a value smaller than some metglasses. At low fields, the torque characteristic of Fe_68.8_Pd_31.2_ exhibits a 2-fold symmetry, which develops into a superposition of 2- and 4-fold symmetry at higher fields. We fitted the data using an *A*sin4(*θ* + *φ*_1_) + *B*sin2(*θ* + *φ*_2_) model and Origin software to look at the relative contribution of each symmetry component to the overall torque, shown in Fig. 3b. A more in-depth look at this process is provided in a supplement. The 2-fold symmetry is a superposition of both the shape anisotropy and an intrinsic 2-fold symmetry. Shape anisotropy accounts for slight differences in the demagnetization factor for orthogonal axes of a non-perfect circle. That is, any amount of ellipticity will contribute a shape anisotropy to the overall system, but for disk-shaped samples it is often negligible. However, Fe_68.8_Pd_31.2_ possesses an unusually low anisotropy energy which means it would be erroneous to discount the contribution of shape anisotropy. We believe this is the case because the response of the amplitude for the 2-fold symmetry as a function of field follows a quadratic pattern, fitted with an adjusted *R*^2^ value of 0.992. This shape anisotropy is independent of temperature, but we also show in Fig. 3c that the amplitude of the 2-fold symmetry has strong temperature dependence, exhibiting a sharp drop before the phase transformation temperature of this material. Such behavior is also seen in a cubic to monoclinic transition for Fe_3_O_4_^25^. Hence we conclude some premartensitic 2-fold crystal symmetry contributes strongly to the magnetic behavior of the system even in the austenitic phase. We assume the remnant 2-fold component of anisotropy at the drop at 271 K accounts for the shape anisotropy of our system, totalling ~120 J/m^3^. This corresponds to a difference of 1.40 × 10^−3^ in the biaxial demagnetization factors, which amounts to a deviation of 0.1% or 5 *μ*m around the perimeter of the disk.

It is unusual for a material to exhibit an anisotropy so highly dependent on the magnetic field, but this can be reconciled by considering the anisotropy is a manifestation of both a regular magnetic component, *K*_*m*_ and a magnetoelastic component *K*_*me*_. At low fields, the magnetoelastic component averages to zero over the bulk of the crystal due to premartensitic twinning, and the latent manifestation of the 4-fold symmetry at saturation signals that the premartensitic microstructure may unfold and return to a cubic structure. Other Fe-based alloys, such as FeAl, FeGe^26^, and FeAlSi^27^ exhibit the same magnetic torque response, hinting that these materials possess similar magnetoelastic behavior. We also show the change in phase angles *φ*_1_ and *φ*_2_ in Fig. 3d as a function of field. The cubic phase angle *φ*_1_ remains constant whereas the tetragonal phase angle *φ*_2_ experiences a continuous shift from 43° to 30° as the field increases from 1000 to 2000 Oe, at which point it becomes constant due to magnetic saturation. This shift is a consequence of the magnetic field rearranging and ordering the premartensitic nano-twinned clusters in the crystal.

### Magnetostriction

**Figure 4:**
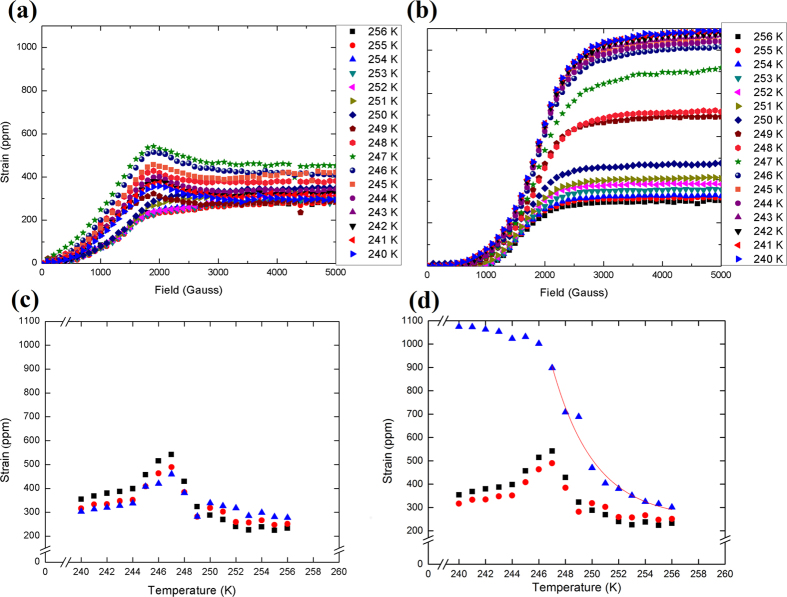
Magnetostriction data shown for single crystal Fe_68.8_Pd_31.2_ at several different temperatures along the (**a**) 〈100〉 and (**b**) 〈110〉 crystalline directions as a function of field. The same data is shown as a function of temperature at select fields ◼ 1900 Oe, ⚫ 2500 Oe, and ▲ 4000 Oe, along the (**c**) 100 and (**d**) 110 crystalline directions. An exponential fit with adjusted *R*^2^ of 0.967 is shown in (**d**) for the 4000 Oe data above 247 K.

The magnetostrictive response of Fe_68.8_Pd_31.2_ is shown in Fig. 4 for a series of temperatures moving through the phase transformation, from 258 to 240 K. Results are shown for orienting single crystals with a strain gauge placed along two perpendicular 〈100〉 directions, with field applied along the [100] and the [110] axes, in Fig. 4a,b, respectively. The plots of Fig. 4c,d show the same results plotted at specific fields as a function of temperature to highlight abrupt changes which occur in the magnetostriction measurements as a result of the phase transformations taking place: from FCC to adaptive FCT to FCT, with transition temperatures at 250 K and 245 K. The slight discrepancy between these temperatures and the transformation temperature from the thermal expansion and magnetic torque data arises from instrumental error, especially for magnetic torque where it is impossible to place the thermocouple on the sample directly. The abrupt transitions apparent in the magnetostriction data affirm that no pseudo-spinodal decomposition takes place within our sample, as in other material systems like (AuCu)-Pt which also possess premartensite phenomena that show a more continuous change instead^28^. However, it can be seen that the values of magnetostriction change close to the transition temperatures, indicating the possibility of short-range strain ordering occurring within the structure in anticipation of the displacive transformation. This is related to distortions caused by nano-platelet precipitates of the tetragonal phase beginning to coalesce and drive the transformation^20^. The data for magnetic saturation shown in Fig. 4d is fitted with an exponential decay function with an adjusted *R*^2^ value of 0.967. The quality of this fit, coupled with the phase shifts in the magnetic torque data, serve as an indication of short-range ordering leading up to the phase transformation.

## Discussion

It was first documented in 1986 by Sugiyama and Oshima^18^ that {110}/〈110〉 striations, ~5 nm apart in TEM transmission mode, appear above the transformation temperature for Fe_68.8_Pd_31.2_, forming an image resembling “tweed” fabric and subsequently earning the name tweed microstructure. This premartensitic phenomenon is related to phonon softening of the [*ξξ*0]TA_2_ mode around the Γ-point near the phase transformation of the material^29^, and the crystalline directions of these striations are attributed to premartensitic nuclei of the tetragonal phase in a weakly first order transformation mechanism^20^. The tweed grows approaching the transformation temperature until a critical cluster concentration is reached, followed by an abrupt transition to a twinned microstructure. Fe_68.8_Pd_31.2_ also exhibits a pseudo-orthorhombic transition phase–similar to that found in Ni_2_MnGa^30^–which exists in a narrow temperature range above *M*_*s*_ and mutually adapts the fully transformed tetragonal and cubic symmetries. Microstructurally this adaptation is accomplished through regular twin modulation by a few interplanar distances: 3:6 in the case of Fe_68.8_Pd_31.2_, as outlined in the theory of Khachaturyan^19^. This adaptive state gives rise to a superlattice reflection in XRD reflecting an orthorhombic symmetry^20^. Recently, Gruner showed that the large degree of twinning on a nanometer scale is attributable to a demagnetization process facilitated by the softening of elastic constants^31^. Gruner’s results also show that the Bain path of the transformation from FCC to FCT structure is a flat energy landscape, which is quantitatively only separated by a barrier of 4 meV/atom. Therefore the tweed structure persists over a large temperature range until the energy is sufficiently large to stabilize the cubic phase.

The elastic softening of Fe_68.8_Pd_31.2_, responsible for both the ease of twinning as well as the low anisotropy energy, also causes the magnetization behavior to mimic the response of amorphous magnetic metals, given by the expression


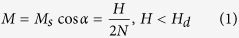


where *M*_*s*_ is the saturation magnetization, *H* is the external field, *N* is the demagnetization factor, and *H*_*d*_ is the demagnetization field, above which the magnetization saturates. Equation (1) is a classical result that reflects pure magnetic domain rotation against the field^32^, and it describes the response of Fe_68.8_Pd_31.2_ very well. The slope of the magnetization curves give a demagnetization factor of 0.071, which matches closely to the theoretical value of 0.081 for our disks with aspect ratio 5:1^33^. This response also resembles that of recently published results on FeGa^16^, where it was demonstrated that the reorientation of twinned demagnetizing cells allows the magnetization to rotate. Gruner’s results^31^ and the similar magnetization behavior suggest that Fe_68.8_Pd_31.2_ magnetizes in a similar manner by unfolding and re-folding twins, though we have not observed any evidence long-range ordering of microscopic twin cells in Fe_68.8_Pd_31.2_.

Since materials which exhibit solely rotational magnetic responses typically possess no crystalline structure, the de-twinning magnetic response of crystalline Fe_68.8_Pd_31.2_ is a mechanism that mimics domain rotation. This is made possible through highly mobile twins in premartensitic tweed clusters which exist to minimize the magnetostatic energy and demagnetize the material when no external field is applied. Without any field applied, the twins within these clusters are arranged such that the average magnetization angle, 〈cos*α*〉, equals 0. Twin rearrangement and detwinning induced by an external field causes this average to deviate between 0 and 1 until saturation, upon which these magnetoelastic twins are eliminated from the clutser and the bulk of the sample fully magnetizes. If each twin is separated by a boundary of *γ*_*me*_ surface energy, one can show the equilibrium number of twins, *n*_0_, in each cluster is


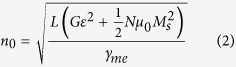


where *L* is a characteristic length of the cluster and *G* is an elastic constant. The system thus consists of two subsets of twins: those that minimize the internal elastic field and those that minimize the internal magnetic field. The process of magnetization eliminates the latter twins from the material, imparting a large magnetoelastic strain into the material.

The isotropic response of the magnetostriction while in the austenitic state is non-Joulian in nature because it violates Joulian conditions that 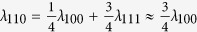, if 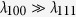 and 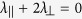
34. This isotropy comes about due to a lack of long-range crystalline ordering in the tweed. It may also be viewed as another symptom of low crystalline anisotropy. However, when the material becomes tetragonal below the transition temperature, the response is no longer isotropic, as seen in Fig. 4c,d. It is atypical for a FSMA to possess such large magnetostriction in the austenitic state as compared to the martensitic state, which we attribute to the premartensitic twin clusters which aren’t found in alloys like Ni_2_MnGa. In addition, we believe the maximum present in Fig. 4c may be a manifestation of magnetic field-induced strains such as those observed in the phase transformation of Ni_2_MnGa^35^. These strains are a small contribution to the overall magnetostriction which arise from classical Joulian formulations of crystal lattice distortion caused by magnetic saturation. They follow an inverse proportionality to the elastic constant *C*′, which causes a peak to occur at the martensitic transformation temperature where the constant tends to zero.

The magnitude of the magnetostriction, ~400 ppm, is comparable to that found by Cui^36^ for a similar FePd alloy under a low state of applied compressive stress. The study makes it clear that the value of 400 ppm magnetostriction represents only a small fraction of the total amount of strain accommodated by twinning during the martensitic transformation, which is ~12000 ppm or 1.2% based on the change in lattice parameters from cubic to tetragonal structure observed in XRD^21^. In that study, it was also shown that a moderate amount of applied stress can suppress the saturated magnetostriction values below the transformation temperature. However, quantitative theoretical frameworks relating the strain one obtains as a result of twin boundary motion from either applied stress or applied magnetic field both remain challenges to formulate in future work.

In summary, we report the existence of large magnetostriction in Fe_68.8_Pd_31.2_ as well as linear, isotropic, and hysteresis free magnetization behavior. This behavior is a consequence of a unique subset of premartensitic magnetoelastic twin clusters able to adapt to low inputs of magnetic or elastic energy without undergoing irreversible changes. Near the martensitic transformation temperature, the change in free energy causes these clusters to condense in a short-range ordering mechanism before transforming into the long-range ordered martensitic state. The results are very similar to those of FeGa as well as FeAl^26^, FeGe^26^, and FeAlSi^27^, indicating these alloys belong to a special class of magnetic materials possessing low effective anisotropy energy, allowing the material to unfold and refold these mobile twins.

## Methods

### Crystal preparation

For our studies, we used single crystal disks Fe_68.8_Pd_31.2_ cut from an ingot that was prepared by arc melting using a high purity Fe rod (99.999%) and a Pd sheet (99.9%) source elements. The single crystal was prepared using a floating zone method. The crystalline orientation was determined by a back-reflection Laue method. The alloy was heat treated at 1373 K for 24 h followed by quenching into ice water in order to manifest martensitic properties which are otherwise metastable at room temperature. The composition, 31.2 atomic % Pd and 68.8 atomic % Fe, corresponds to a minimum of the solid-liquid characteristics thereby suppressing solidification induced inhomogeneities. Disks of 5 mm diameter and 1 mm thickness were cut with both [100] and [110] normals.

### Characterization techniques

Magnetization curves and magnetic torque measurements were taken using a Lakeshore vibrating sample magnetometer. The [110] sample was mounted to magnetize in the plane of the disk for the magnetization, measuring first along the [001] easy axis, then rotated to measure along the [1
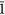
1] then [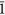
10]. For magnetic torque, a sample is placed in a mount which can rotate within the field and detect the force needed to complete such rotation. Each torque measurement has the field applied in plane, starts at the [1
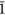
1] direction, and rotates about the [110] normal of the disk. Strain measurements were achieved using a quarter-Wheatstone bridge method with gauges adhered using an M100 bond epoxy from Vishay Measurements Group. The gauges were stacked tee rosette designs of 120 Ω resistance from the same company. The samples were then mounted on a Peltier device temperature-controlled stage in a vacuum of approximately 10^−3^ torr. Thermal expansion measurements were taken using this stage between temperatures of 230 K to 325 K, and magnetostriction measurements were taken while suspending this stage in an electromagnet.

## Additional Information

**How to cite this article**: Steiner, J. *et al.* Unique magnetostriction of Fe_68.8_Pd_31.2_ attributable to twinning. *Sci. Rep.* **6**, 34259; doi: 10.1038/srep34259 (2016).

## Supplementary Material

Supplementary Information
