# Supplementary material for: Unique magnetostriction of Fe68.8Pd31.2 attributable to twinning
Source: Sci Rep. 2016 Sep 30;6:34259. doi: 10.1038/srep34259 (PMC5043241; doi:10.1038/srep34259)

Unique magnetostriction of $Fe_{68.8}Pd_{31.2}$ attributable to twinning

Jake Steiner [1,*] , *Abdellah Lisfi*[2], *Tomoyuki Kakeshita*[3], *Takashi Fukuda*[3], *and Manfred Wuttig*[1]

[1]University of Maryland, Department of Materials Science and Engineering, College Park, MD 20902, USA

[*]corresponding author, jrstein92@gmail.com

[2]Morgan State Univeristy, Department of Physics, Baltimore, MD 21251, USA

[3]Osaka University, Department of Materials Science and Engineering, Suita, Osaka 565-0871, Japan

S1. Deconvoluting the Magnetic Torque of $Fe_{68.8}Pd_{31.2}$

This supplement provides a better look at individual magnetic torque curves for the main paper to illustrate the 2-fold and 4-fold symmetry that exists within the crystal at different field strengths. All data taken was fit using an $A \sin 4(\theta + \varphi_1) + B \sin 2(\theta + \varphi_2)$ in Origin software. The following plots will show the ■raw data of each measurement, a (red) fitted line obtained using this model to the data, as well as the waveforms of the (blue) 2-fold component, $B \sin 2(\theta + \varphi_1)$, and (orange) 4-fold component, $A \sin 4(\theta + \varphi_1)$ for illustrative clarity.

300 Oe Torque

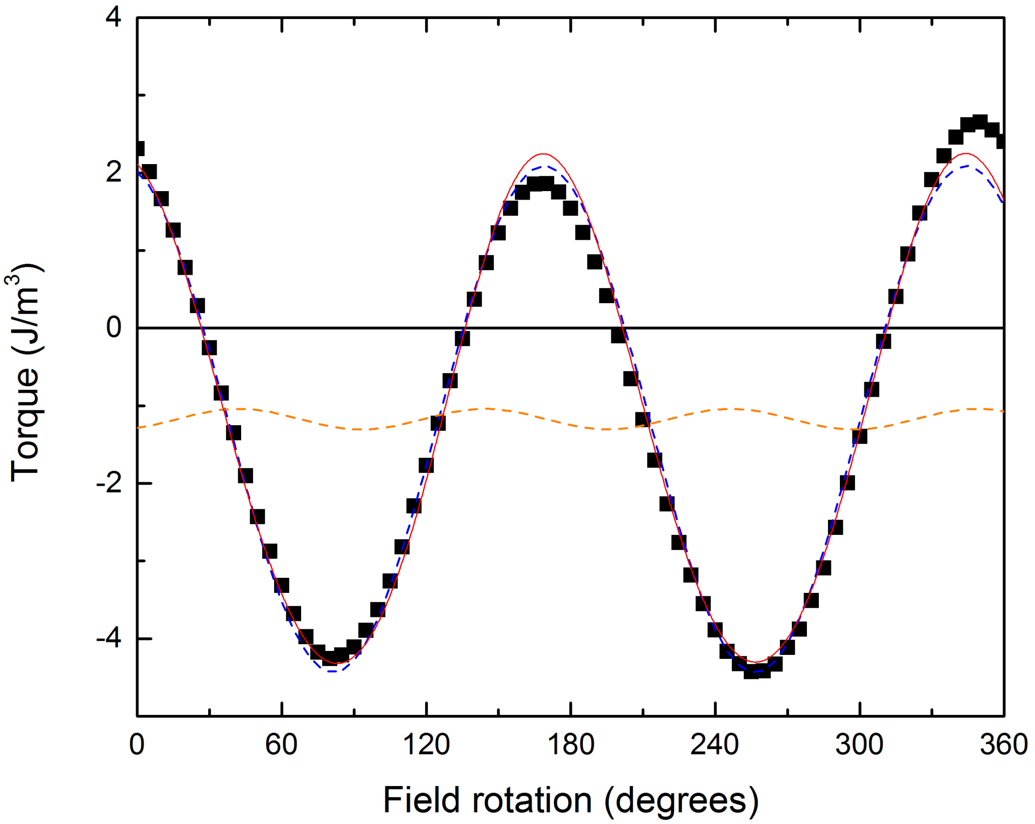

1200 Oe Torque

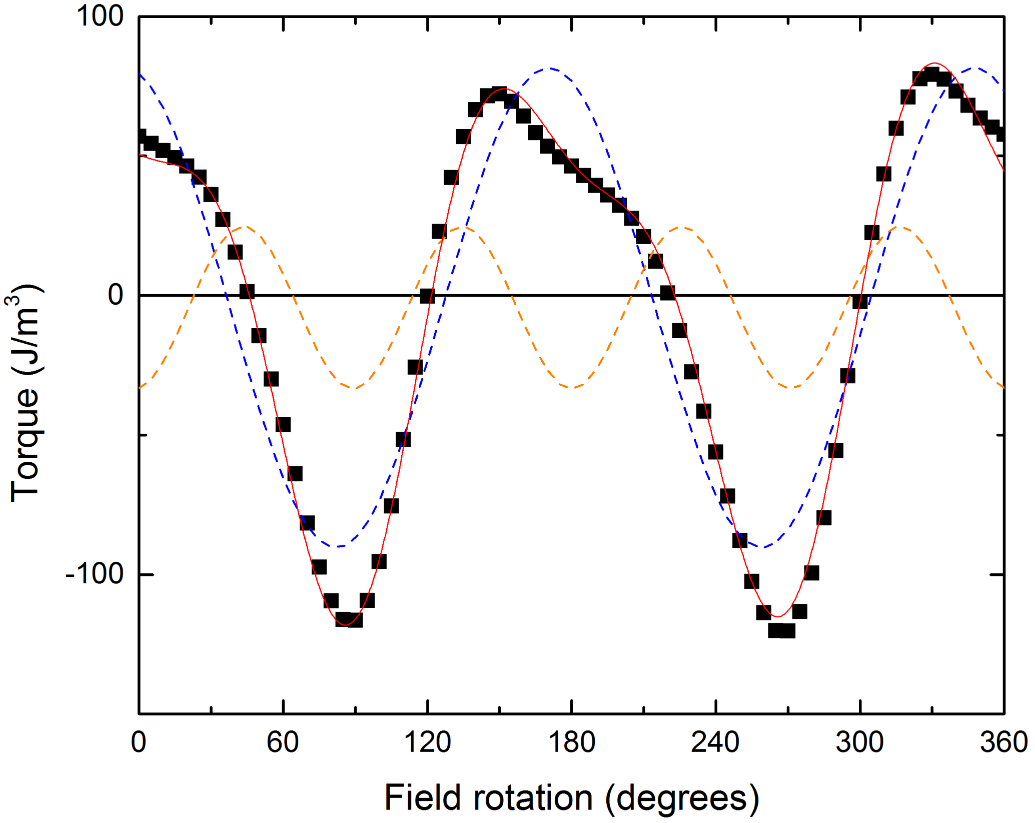

3000 Oe Torque

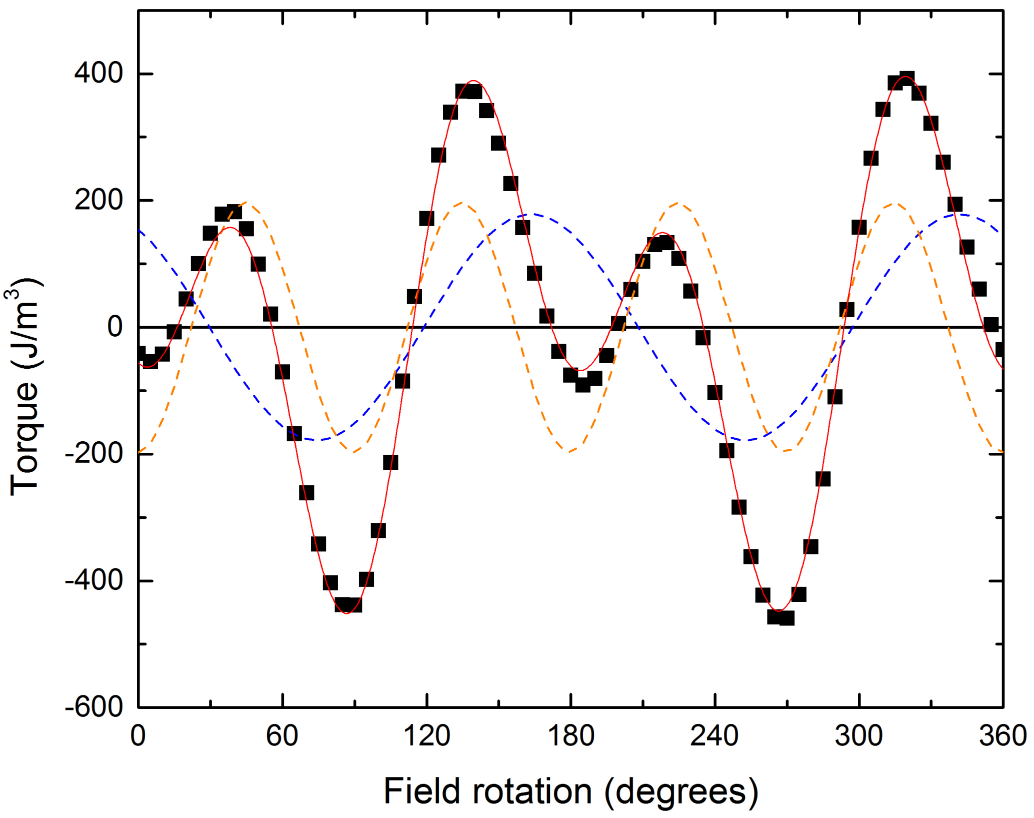

Supplement: Supplementary Information [file srep34259-s1.pdf]
